# Identification of PANoptosis-related biomarkers in myocardial infarction via bioinformatics and single-cell analyses

**DOI:** 10.1097/MD.0000000000046750

**Published:** 2026-01-23

**Authors:** Dongdong Que, Pengfei Nie

**Affiliations:** a Department of Cardiovasology, ZhuJiang Hospital of Southern Medical University, Guangzhou, Guangdong Province, China; b Department of Cardiovasology, Guangzhou Hospital of Integrated Traditional and Western Medicine, Guangzhou, Guangdong Province, China; c The First Affiliated Hospital of Guangzhou University of Chinese Medicine, Guangzhou, Guangdong Province, China.

**Keywords:** bioinformatics, inflammation, myocardial infarction, PANoptosis, single-cell RNA-sequencing

## Abstract

The relevance of PANoptosis, a new programmed cell death discovered recently, to myocardial infarction (MI) is unknown. This study aims to identify biomarkers associated with PANoptosis in MI and pathogenic mechanisms. Differentially expressed genes (DEGs) of MI were obtained firstly. PANoptosis-related genes (PRGs) were obtained from the Genecard database. PANoptosis-related DEGs (PRDEGs) were obtained by taking a cross between DEGs and PRGs. Functional enrichment analysis was performed on PRDEGs. Single-cell analysis was performed to identify cell clusters related to PANoptosis. After merging DEGs and PRGs, 3 candidate genes were obtained: IL-1β, NLRP3 and toll-like receptor 4 . Both nomogram and receiver operating characteristic curves showed good clinical efficacy for all 3 candidate genes. Enrichment analysis indicated that these 3 genes are participating in inflammatory pathways including NF-κB. Single-cell analysis revealed that these 3 genes were predominantly expressed in monocytes/macrophages. Monocytes/Macrophages from MI patients and controls were differently distributed on the differentiation trajectories of the pseudotime analysis. Monocytes/Macrophages from MI patients were less likely to be involved in cardiac muscle tissue morphogenesis, muscle tissue morphogenesis, and other processes. Monocytes/Macrophages are associated with PANoptosis in MI. And NLRP3, IL-1β, and toll-like receptor 4 may be important biomarkers for this process.

## 1. Introduction

As society develops and living habits change, cardiovascular disease (CVD) has become a major reason for death in China.^[1]^ Acute myocardial infarction (MI), as an acute and serious condition in CVD, accounts for around 60% of death from CVD.^[2]^ Although advancements in revascularization therapies have improved short-term survival rates, persistent challenges including ischemia-reperfusion injury and coronary restenosis continue to compromise long-term outcomes.^[3,4]^ A better understanding of the mechanism of MI can be beneficial for both preventive strategies and therapeutic innovations.

Programmed cell death (PCD), first named in 1972,^[5]^ has evolved to encompass distinct yet interconnected pathways including apoptosis, pyroptosis, and necroptosis.^[6,7]^ Emerging evidence reveals intricate crosstalk among these pathways. For instance, caspase-8 (CASP8) serves as a shared regulator of apoptosis and necroptosis,^[8]^ while caspase-3 (CASP3) cleaves gasdermin E (GSDME) to induce pyroptosis in multiple cell types.^[9]^ Furthermore, necroptosis triggers NLRP3 inflammasome activation through MLKL pore-mediated potassium efflux in a cell-intrinsic manner,^[10]^ functionally linking necroptosis to pyroptosis. These interactions culminated in the discovery of PANoptosis – a coordinated cell death modality orchestrated by multiprotein PANoptosome complexes that simultaneously engage pyroptotic, apoptotic, and necroptotic machinery.^[11–13]^ An animal experiment has shown that inhibition of key proteins in pyroptosis, apoptosis, and necroptosis alone does not alleviate the disease, while deleting molecules from all 3 pathways simultaneously take effect.^[14]^ Studies have shown that MI may induce apoptosis, pyroptosis, and necroptosis,^[15–17]^ but there are few studies on PANoptosis after MI.

The inflammatory axis bridges the pathogenesis of PANoptosis and MI. Atherosclerosis (AS) is the main pathological manifestation of MI. The inflammatory theory of AS formation was first proposed in 1999 and has been increasingly refined.^[18]^ Inflammation is not only involved in disease formation, but after MI occurs, excessive inflammatory response with cardiac fibrosis may trigger arrhythmias, heart failure and other complications.^[19]^ It has been well reported that NLRP3 inflammasome is implicated in the inflammatory response after MI and may contribute to adverse cardiac remodeling.^[20–22]^ Clinical trials suggest that controlling inflammation contributes to the prevention and treatment of AS.^[23,24]^ Interleukin-1β (IL-1β) inhibitors can reduce high-sensitivity C-reactive protein (hsCRP) levels and lower the incidence of cardiovascular events in patients with chronic kidney disease.^[25]^ Inflammatory responses are also central to microvascular dysfunction in MI. Recent clinical studies highlight the systemic immune-inflammation index (SII) – a composite marker of neutrophil, platelet, and lymphocyte counts – as a predictor of ischemia in patients with nonobstructive coronary arteries.^[26]^ This underscores how systemic inflammation may drive ischemic injury even in the absence of overt coronary stenosis, emphasizing its broader role in MI pathophysiology. Similarly, emerging inflammatory indices such as the uric acid/albumin ratio have demonstrated prognostic utility in predicting post-MI complications, further validating the systemic inflammatory axis as a critical driver of cardiovascular outcomes.^[27]^ PANoptosis was initially characterized in microbial infections,^[13,28,29]^ while recent work links it to sterile inflammatory diseases such as autoimmune disorders.^[30]^ All the 3 PCD modalities exhibit bidirectional interactions with inflammation.^[31–33]^ Systemic inflammation post-MI may also drive PANoptosis activation, amplifying myocardial injury and fibrosis. This knowledge gap acquires significance in light of recent findings that doxorubicin-induced cardiomyopathy involves PANoptotic activation in cardiomyocytes,^[34]^ suggesting potential mechanistic parallels in ischemic heart disease.

**Figure 1.**
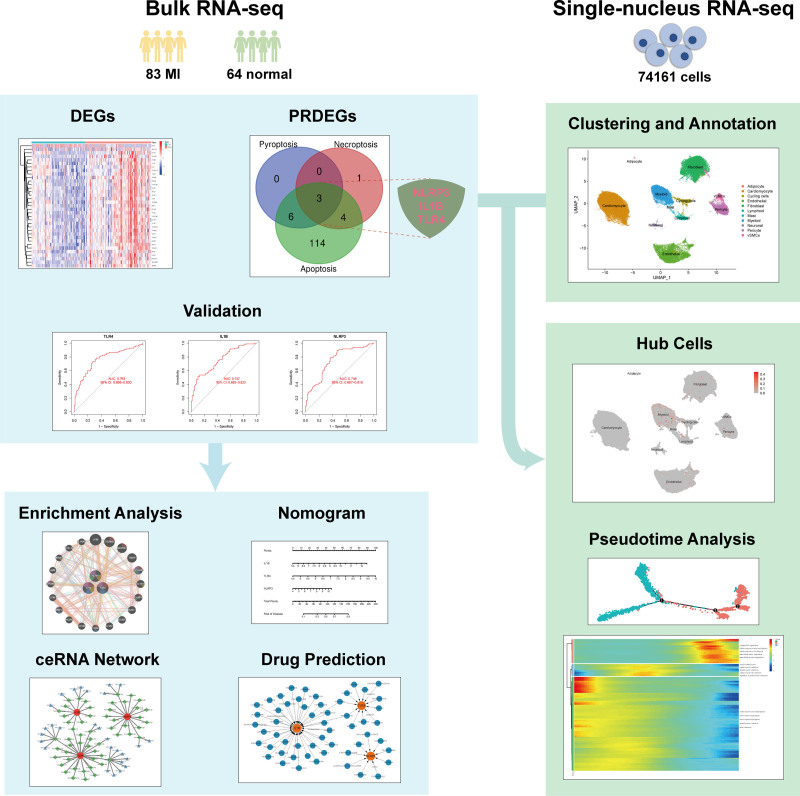
Flowchart of the study.

To investigate this hypothesis, we employ an integrative bioinformatics approach combining bulk tissue analysis with single-cell resolution studies. This dual strategy enables both systemic-level biomarker discovery and cellular mechanistic exploration, capitalizing on the complementary strengths of population-level gene expression patterns and cell-type-specific pathway activation. The workflow of this study is shown in Figure 1.

## 2. Materials and methods

### 2.1. Data access

Through the gene expression omnibus database (https://www.ncbi.nlm.nih.gov/geo/), we had access 4 MI bulk RNA-sequencing (RNA-seq) datasets [GSE48060,^[35]^ GSE60993,^[36]^ GSE61144,^[36]^ GSE66360^[37]^]. 1 MI single-nucleus RNA-seq (snRNA-seq) dataset^[38]^ was obtained from CELLxGENE database (https://cellxgene.cziscience.com/). The source species for the datasets are all *Homo sapiens*. The details of the characteristics of the 4 datasets were shown in the Table S1, Supplemental Digital Content, https://links.lww.com/MD/R99.

### 2.2. Identification of DEGs and PRDEGs

Four bulk RNA-seq datasets were merged using R software. With the “limma” package, the differentially expressed genes (DEGs) between MI and normal samples in datasets were identified.

To delineate PANoptosis-related mechanisms, apoptosis-, pyroptosis-, and necroptosis-related genes were collected from 2 complementary sources: KEGG (https://www.genome.jp/kegg/) and GeneCards (https://www.genecards.org/), retaining genes with relevance scores > 0.5. For each PCD modality (apoptosis, pyroptosis, and necroptosis), the corresponding gene sets were independently cross-referenced with DEGs filtered under relaxed thresholds (adjusted *P* < .05 and |log_2_ FC| > 0.2). Finally, the overlapping genes across all 3 PCD modalities were defined as PANoptosis-related DEGs (PRDEGs).

### 2.3. Validation and nomogram for PRDEGs

The expression levels of PRDEGs in the disease and control groups were visually compared by box plots. The receiver operating characteristic analysis was applied to test the clinical reliability of PRDEGs.

Based on PRDEG, a nomogram was created utilizing the “rms” package. The accuracy of the nomogram was assessed by calibration curves. A decision curve analysis and clinical impact curves (CIC) were performed to evaluate the clinical utility of the nomogram.

### 2.4. Protein–protein interaction network and functional enrichment analysis

The Genemania (http://www.genemania.org) is an online tool to construct protein–protein interaction (PPI) networks for a given list of genes,^[39]^ which was used to build a PPI network for PRDEGs. Then, gene ontology functional enrichment analysis was applied to identify the molecular function, cellular component, and biological process associated with PRDEGs.

### 2.5. Establishment of ceRNA network

The competing endogenous RNA (ceRNA) network was constructed through a multi-tiered bioinformatics workflow. First, miRNA-mRNA interactions were predicted using miRDB^[40]^ (http://mirdb.org) with stringent miRanda algorithm parameters (binding score ≥ 80) and experimentally validated against miRTarBase^[41]^ (miRTarBase.cuhk.edu.cn/), retaining only interactions supported by CLIP-seq or luciferase reporter evidence. Next, starBase^[42]^ was employed to identify lncRNA-miRNA pairs meeting thermodynamic stability criteria (minimum free energy ≤ -20 kcal/mol) with seed region complementarity. High-confidence tripartite relationships were established by linking lncRNAs sharing ≥ 3 common miRNAs across PANoptosis-related DEGs (PRDEGs) using the R package “igraph.”

### 2.6. Prediction of drug

Based on the DGIdb database^[43]^ (dgidb.org/), potential drugs that may act on the target genes was screened. And the network of genes and drugs was constructed.

### 2.7. SnRNA-seq data processing

Single-nucleus RNA-sequencing (snRNA-seq) data were processed using the “Seurat”^[44]^ package in R. Initial quality control retained cells expressing 200-4000 unique genes with mitochondrial gene content < 10%, excluding low-quality or apoptotic cells. Following normalization and scaling, 2000 highly variable genes were selected using variance-stabilizing transformation for downstream analysis. Principal component analysis was performed on highly variable genes, followed by batch correction using “Harmony”^[45]^ package to mitigate inter-sample variability. Cell clusters were identified via shared nearest neighbor graph construction (k = 20 neighbors) and Louvain algorithm clustering (resolution = 0.8), visualized using uniform manifold approximation and projection embeddings.

### 2.8. Mannual annotation

Cluster-specific marker genes were identified using the “FindAllMarkers” function in “Seurat” package with Wilcoxon rank-sum test (adjusted *P* < .01, |log_2_FC| > 1, minimum cell fraction > 20%). The top 10 DEGs per cluster were cross-referenced against the CellMarker database^[46]^ (http://bio-bigdata.hrbmu.edu.cn/CellMarker), which consolidates experimentally validated markers from over 200,000 published single-cell studies. Consensus cell identities were visualized on uniform manifold approximation and projection plots.

### 2.9. Cell–cell Communication analysis

Cell–cell communication networks were systematically profiled using the “CellChat” package. First, normalized expression matrices were preprocessed to identify overexpressed ligands/receptors (mean expression > 0.1 in ≥ 10% cells per cluster). Communication probabilities were computed via “computeCommunProb” and “computeCommunProbPathway” functions. Differential interaction strengths were quantified through generalized linear models adjusted for cellular composition covariates, with results visualized as hierarchically clustered heatmaps using the “netVisual_heatmap” function.

### 2.10. Identification of hub cells and pseudotime analysis

The expression levels of PRDEGs in various cell clusters were evaluated using the “AddModuleScore” function. We suggested that hub cell cluster with higher expression levels may be more involved in the process of PANoptosis. Since myeloid cells were the more highly expressed cells, data of myeloid cells were extracted. Similarly, using “FindVariableFeatures,” “FindNeighbors,” and “FindClusters” functions, myeloid cells were reclustered. The “singleR” package was used for annotation. Monocytes/Macrophages were the main component of myeloid cells. Then, a pseudotime analysis of the monocytes/macrophages was performed with the “monocle” package to generate a pseudotime trajectory.

Moreover, the “FindMarkers” function was employed to locate DEGs of monocytes/macrophages between patients and controls. Changes in the expression of DEGs in the pseudotime trajectory were shown using heatmaps via the “monocle” package. The NetworkAnalyst platform^[47]^ (https://www.networkanalyst.ca/) was applied to find reliable transcription factors (TFs) that bind to hub genes from the ENCODE database. Changes in the expression of TFs was also presented with heatmap.

## 3. Results

### 3.1. DEGs of MI

**Figure 2.**
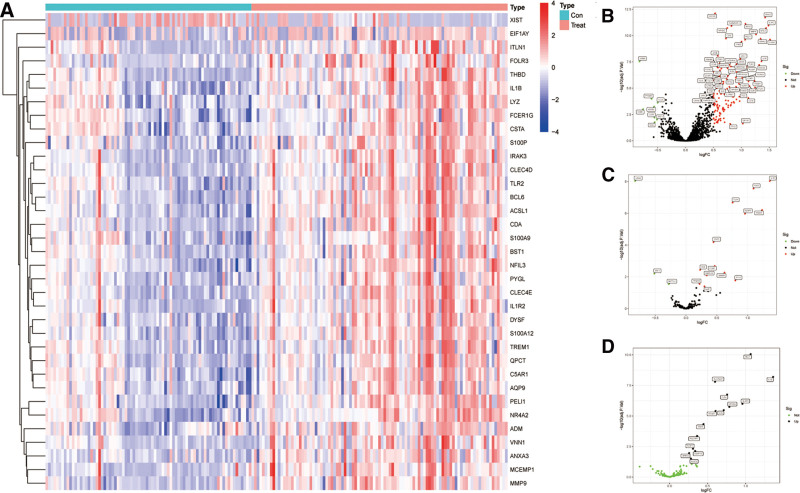
(A) Heatmap of 36 DEGs betweens MI and controls in dataset GSE48060, GSE60993, GSE61144, GSE66360. Red: upregulated; blue: downregulated. (B–D) Volcano plot of apoptosis-related, pyroptosis-related, and necroptosis-related DEGs. DEGs = differentially expressed genes, MI = myocardial infarction.

36 DEGs was identified between patients with MI and controls, the expression of DEGs was visualized with heatmap (Fig. 2A). The intersection of all DEGs with the 3 patterns of PCD (apoptosis, necroptosis, pyroptosis) -related genes were taken separately, each screened with criterion of *P* < .05 and |log_2_ FC| > 0.2. The volcano plots showed the upregulated and downregulated expression of the most significant PCD-related DEGs (|log_2_ FC| > 2), respectively (Fig. 2B–D).

### 3.2. 3 PRDEGs were upregulated in MI patients

**Figure 3.**
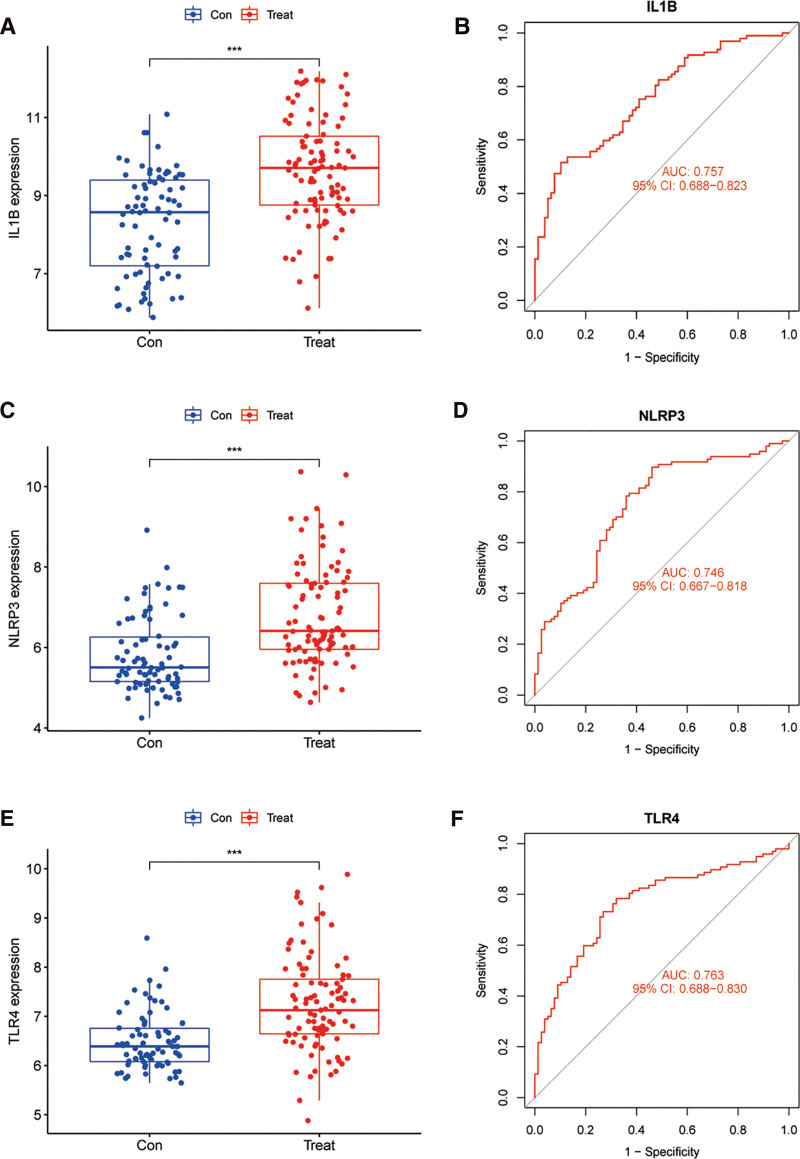
(A, C, E) The boxplot of IL-1β, NLRP3, and TLR4 expression in patients with MI (red) and controls (blue). (B, D, F) The ROC curve of IL-1β, NLRP3, and TLR4. (***: *P* < .001). MI = myocardial infarction, TLR4 = toll-like receptor 4.

After identifying the intersection of apoptosis-, pyroptosis-, and necroptosis-related DEGs, we got 3 hub genes. These 3 hub genes were IL-1β, NLRP3, and toll-like receptor 4 (TLR4), which were PANoptosis-related DEGs in MI patients. The expression of 3 hub genes in patients with MI and control was visualized with box plot (Fig. 3A, C, E). All 3 hub genes shared an upward trend of expression in the MI group. And all the differences between the MI group and the control group were statistically significant (*P* < .001). Receiver operating characteristic curves were also generated for them to validate the diagnostic ability (Fig. 3B, D, F), with an area under the curve of 0.757, 0.746, and 0.763 in the training dataset. These AUC values demonstrate moderate but consistent discriminative capacity in distinguishing MI patients from controls, suggesting their potential utility as a combinatorial biomarker panel for early MI detection.

### 3.3. 3 PRDEGs with good efficacy in predicting disease

**Figure 4.**
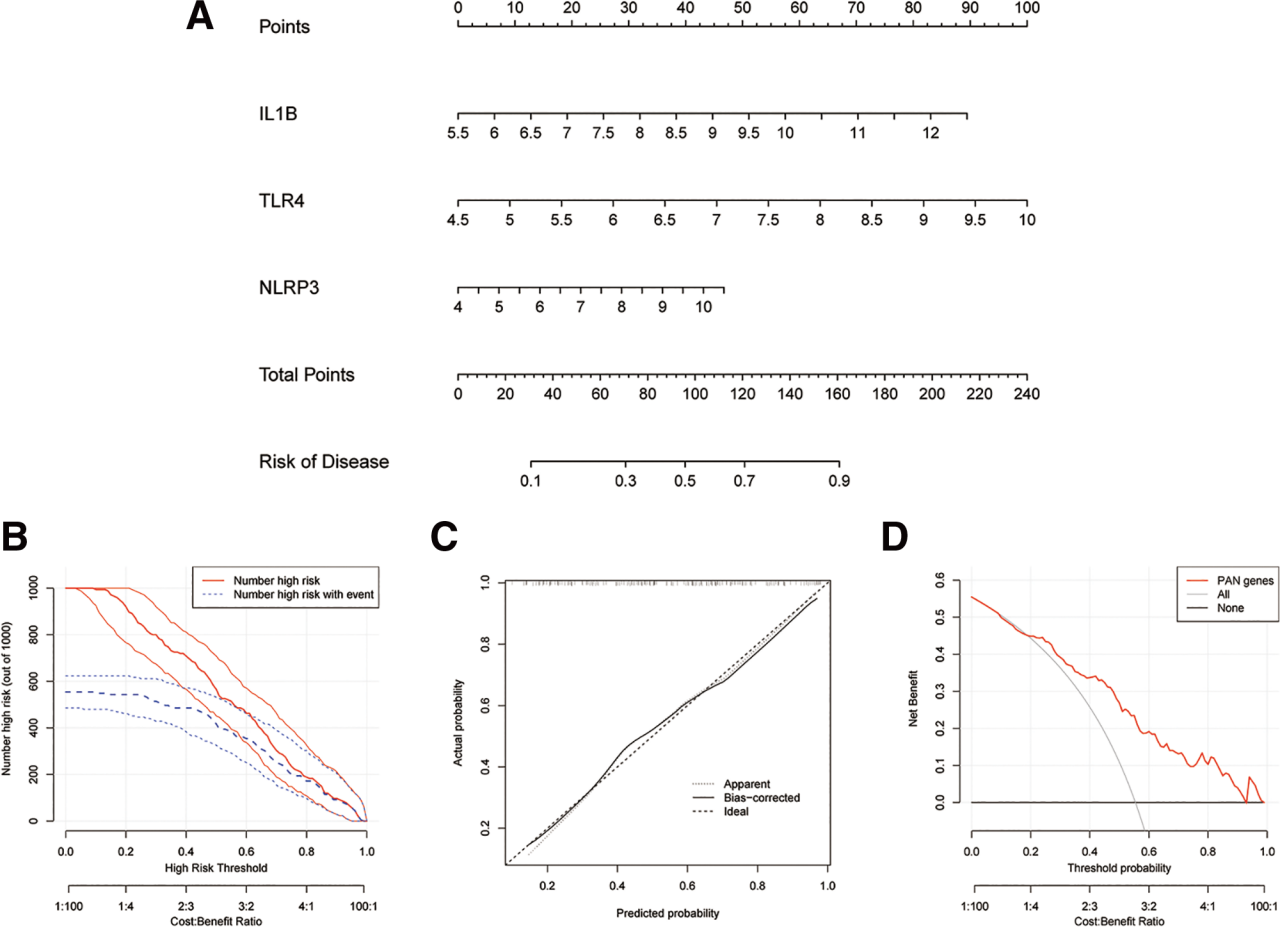
(A) A nomogram predicting the risk of MI based on IL-1β, TLR4 and NLRP3. (B) Clinical impact curve of the nomogram. The red curve indicates the number of people who are positives (high risk) in the model. The blue curve indicates the number of true positives (high risk of an event). (C) The calibration curves of the nomogram. X-axis represents the nomogram-predicted probability. Y-axis represents the actual probability of invasive adenocarcinoma. (D) Decision curve analysis of the nomogram. X-axis indicates the threshold probability for MI and Y-axis indicates the net benefit. MI = myocardial infarction, TLR4 = toll-like receptor 4.

A nomogram that intergrated 3 PRDEGs for the incidence of MI is shown in Figure 4A. CIC was used to evaluated clinical applicability of nomogram (Fig. 4B). The CIC shows that the nomogram predicted a higher number of diseases than the actual number of events within a wide range of risk thresholds. And the difference was more pronounced at low risk thresholds, suggesting significant predictive value for the 3 hub genes. The calibration curve shows a high consistency between prediction and actual observation (Fig. 4C). According to the decision curve analysis shown in Figures 4D, 3 PRDEGs had a good net benefit, which also indicated that the 3 PRDEGs performed well in preditcting the incidence of MI.

### 3.4. PPI network and function enrichement analysis of 3 PRDEGs

**Figure 5.**
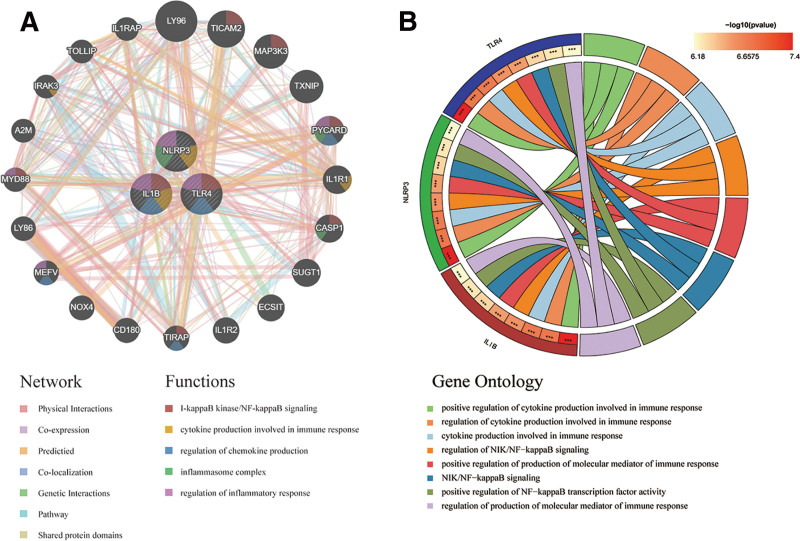
(A) PPI network of 3 hub genes. The surrounding dots are the 20 most relevant genes to 3 key genes. The colors of the connecting lines represent different networks. The colors in the dots represent the functions in which the genes are involved. (B) Circle plot of GO analysis of 3 hub genes. GO = gene ontology, PPI = protein–protein interaction.

Based on the GeneMania database, a PPI network was constructed for 3 PRDEGs to analyze their functions (Fig. 5A). Color of dots in the network indicated that 3 key genes were involved in multiple inflammatory responses and pathways, such as NF-κB signaling, cytokine production involved in immune response, and regulation of inflammatory response. We also performed gene ontology analysis on 3 PRDEGs to explore the biological functions and pathways involved (Fig. 5B). The results similarly showed that 3 PRDEGs were associated with multiple inflammatory responses and pathways including cytokine production involved in immune response and regulation of NF-κB signaling.

### 3.5. Identification of the potential drugs

**Figure 6.**
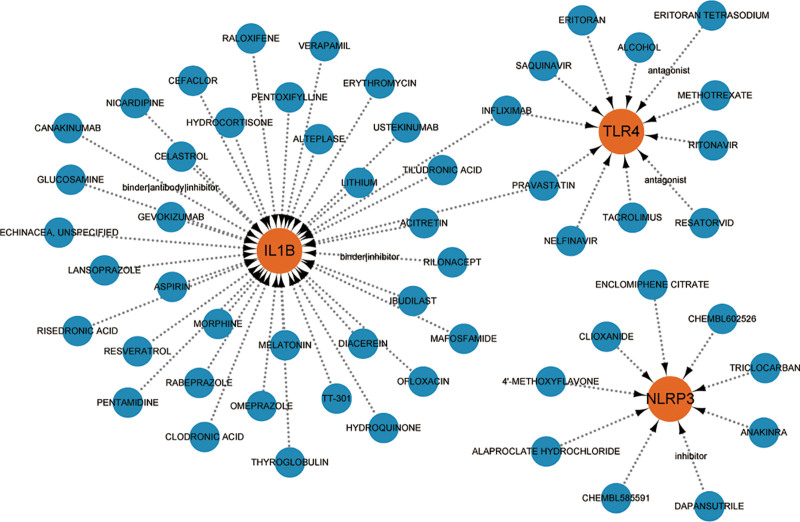
Gene-drug interaction network. Orange dots are the genes, and the blue dots are the drugs.

Based on the DGIdb, potential drugs or molecular components that may regulate the expression of 3 hub genes were identified. As shown in the interaction network (Fig. 6), 37 drugs or molecular components including acitretin, lithium, and aspirin regulate IL-1β. 10 drugs or molecular components including pravastatin, eritoran, and saquinavir were found to interact with TLR4. 9 drugs or components that included anakinra, triclocarban, and clioxanide regulated NLRP3. Notably, infliximab and pravastatin may have effects on both IL-1β and TLR4.

### 3.6. Construction of ceRNA network

**Figure 7.**
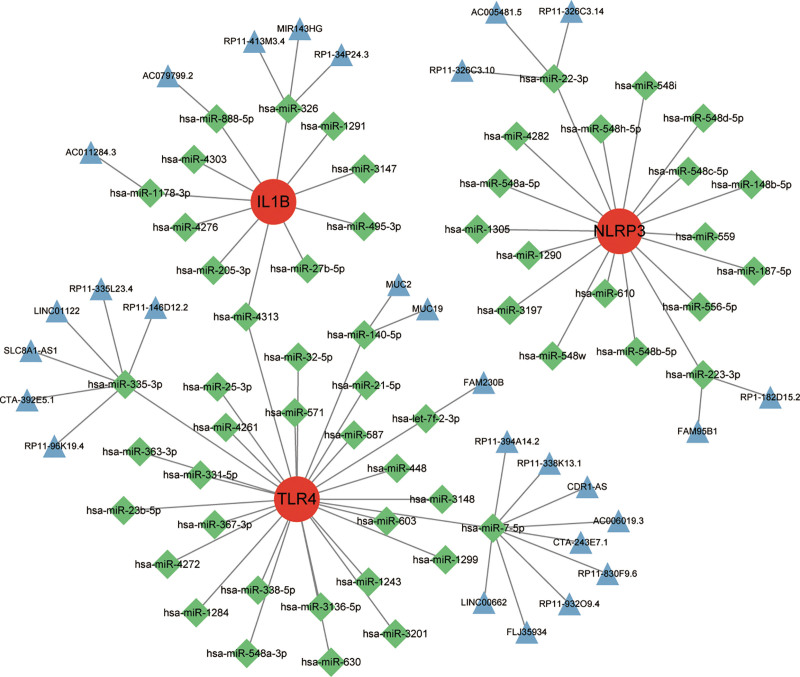
ceRNA network of 3 hub genes. Red dots are mRNAs, green squares are miRNAs, blue triangles are lncRNAs.

Through miRDB, miRTarBase, and starBase databases, a ceRNA network was constructed (Fig. 7). In total, 54 miRNA nodes and 27 lncRNA nodes were identified. Three genes are connected to numerous miRNAs, suggesting potential regulatory relationships. For instance, IL-1β is linked to miRNAs such as hsa-miR-885-5p, hsa-miR-4303, and hsa-miR-1178-3p, while TLR4 is associated with miRNAs like hsa-miR-335-3p, hsa-miR-25-3p, and hsa-miR-32-5p. Notably, hsa-miR-4313 is identified as a potential regulator of both IL-1β and TLR4, indicating its significant role in modulating these genes’ expression.

However, hsa-miR-335-3p and hsa-miR-7-5p are found to compete with multiple lncRNAs for target binding. This competition implies that these miRNAs may not be ideal candidates for precisely regulating the expression of corresponding genes. Their interactions with various lncRNAs could lead to less specific or less efficient regulation of target genes like IL-1β and TLR4, complicating their role in therapeutic or experimental settings.

### 3.7. Clustering and annotation of snRNA-seq dataset

**Figure 8.**
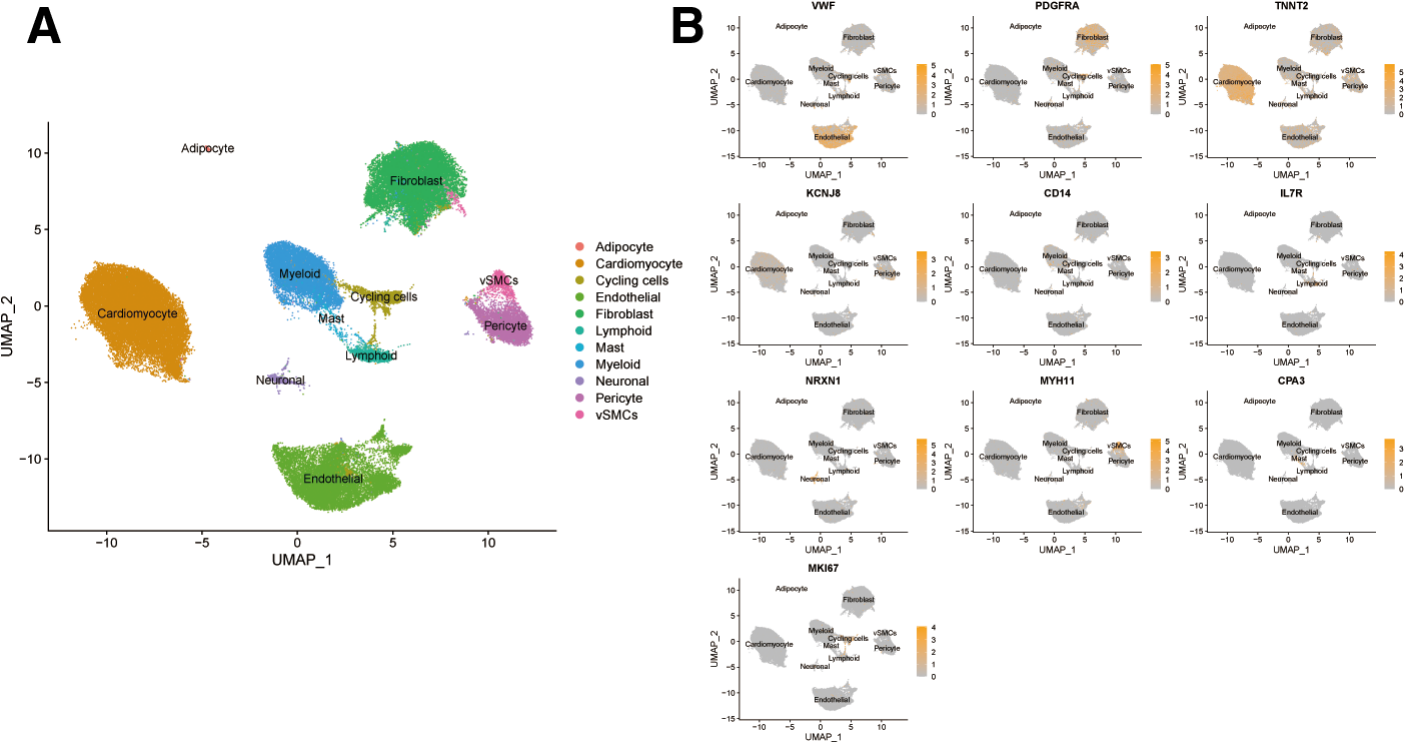
(A) Annotation of cell clusters in MI dataset. (B) Feature plot of markers in cell clusters. MI = myocardial infarction.

After quality control, clustering and annotation, cells in the MI snRNA-seq dataset were categorized into 11 cell clusters, including adipocyte, cardiomyocyte, cycling cells, endothelial, fibroblast, lymphoid, mast, myeloid cells, neuronal, pericyte, and vascular smooth muscle cells (vSMCs) (Fig. 8A). Markers were applied for the annotation of cell clusters, such as VMF for endothelial, PDGFRA for fibroblast, and TNNT2 for cardiomyocyte. All markers were highly expressed in specific cell clusters (Fig. 8B).

### 3.8. Cell–cell communication network

**Figure 9.**
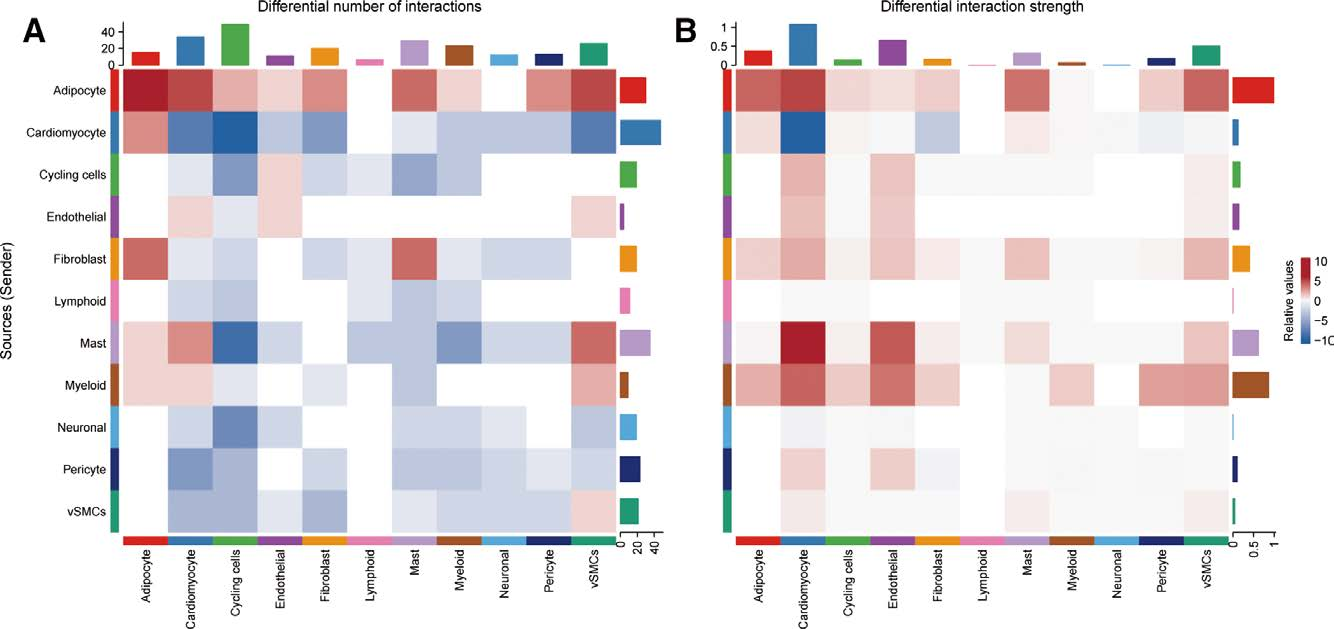
(A) Heatmap shows the relative number of interactions of cell clusters in the MI group compared with the control group. (B) Heatmap shows the relative strength of interactions of cell clusters in the MI group compared with the control group. MI = myocardial infarction.

As shown in heatmaps (Fig. 9), cell–cell interactions in patients with MI were significantly changed from normal controls. Adipocytes had more interactions with other cell clusters than before. And cardiomyocytes had fewer interactions with other cell clusters, which may be related to myocardial ischemic necrosis after MI. As for the intensity of interactions, myeloid cells, as source cells, interacted with cardiomyocytes and fibroblasts more than before, which may be related to the release of inflammatory factors from macrophages after the inflammatory response in MI.

### 3.9. Identification of PANoptosis-related cells and pseudotime analysis

**Figure 10.**
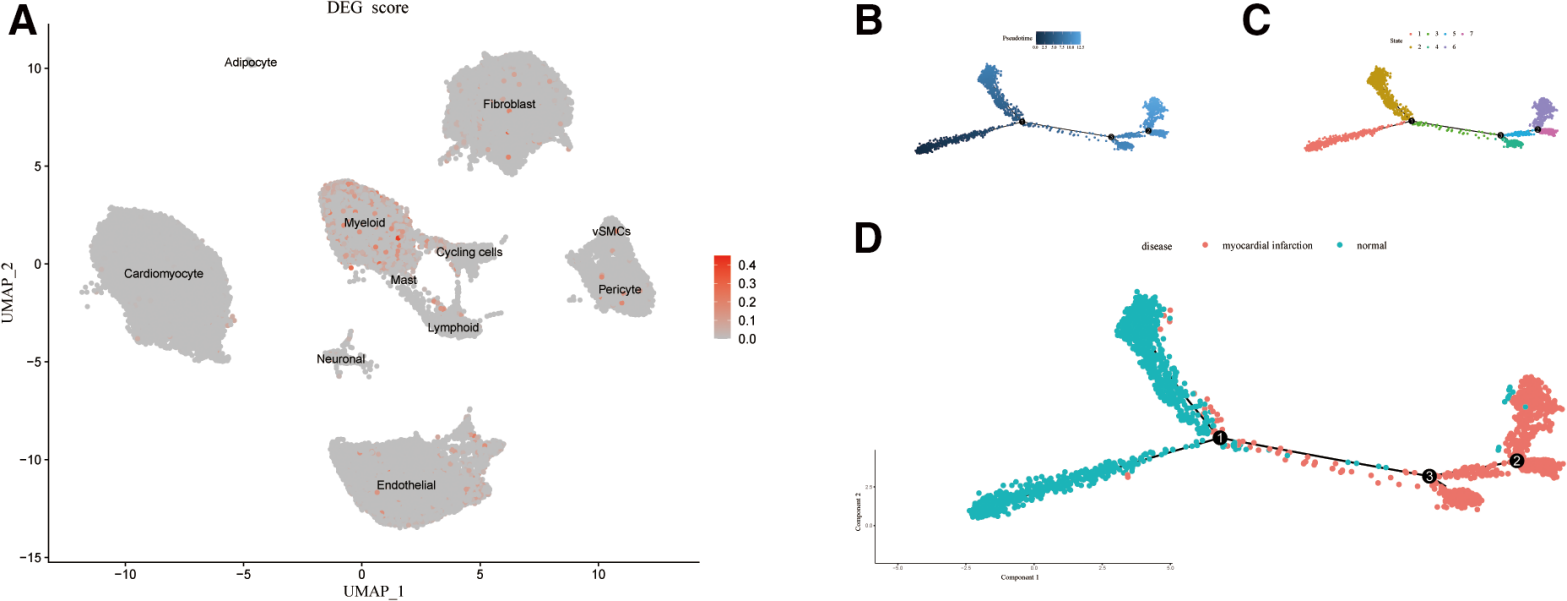
(A) PANoptosis-related DEGs score of all cell clusters. Red dots indicate higher scores. (B–D) Pseudotime analysis of MI scRNA-seq dataset. (B) Differentiation trajectories of macrophages in pseudotime analysis. The chronological order was from left-to-right and the color changed from dark blue to light blue. (C) Multiple states of macrophages in differentiation trajectories. (D) Distribution of macrophages in differentiation trajectories in MI patients and controls. Green was the control group and red was the MI group. DEGs = differentially expressed genes, MI = myocardial infarction.

After scoring the expression of the 3 hub genes, we found that these genes were highly expressed in myeloid cells compared to other cell cluters (Fig. 10A). This suggested that myeloid cells may be more involved in PANoptosis. We extracted data of myeloid cells for reclustering and annotation, and monocytes/macrophages accounted for the vast majority of these myeloid cells (Fig. S1, Supplemental Digital Content, https://links.lww.com/MD/R99). Then, these monocytes/macrophages were used to perform the pseudotime analysis (Fig. 10B–D). As shown in Figure 10B, monocytes/macrophages differentiate on a left-to-right trajectory, changing from dark blue to light blue. Monocytes/Macrophages on the trajectory were categorized into 7 states (Fig. 10C). There was a significant difference between monocytes/macrophages from MI patients and controls, and the distribution of both on the differentiation trajectory was shown in Figure 10D. This suggests that the major subtypes of monocytes/macrophages in MI patients differ from those in controls.

### 3.10. Pathways and TFs expression changes during pseudotime analysis

**Figure 11.**
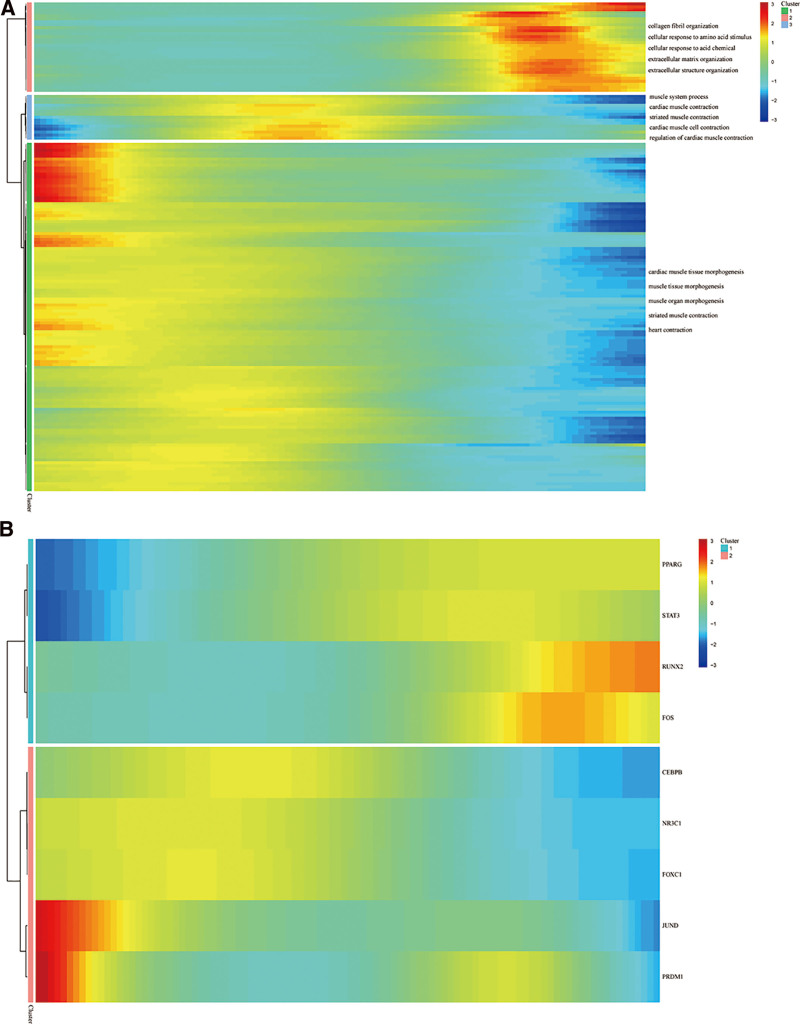
(A) Heatmap of genes expression in pseudotime trajectory. (B) Heatmap of expression of hub genes related TFs in pseudotime trajectory. TFs = transcription factors.

Utilizing the “FindMarkers” function, we obtained 161 DEGs of monocytes/macrophages from MI patients versus controls (Table S2, Supplemental Digital Content, https://links.lww.com/MD/R99). To investigate the biological processes that macrophages were involved in after the onset of MI, we clustered 161 DEGs according to the differences in expression trends in pseudotemporal analysis. All DEGs were categorized into 3 clusters, with the expression of the first group of DEGs gradually increasing, the second group increasing and then decreasing, and the third group gradually decreasing (Fig. 11A). Genes with progressively higher expression are involved in collagen fibril organization, cellular response to amino acid stimulus, cellular response to acid chemicals, extracellular matrix organization, and extracellular matrix structural organization of these biological processes. It is suggested that macrophages in MI patients may be involved in these biological processes. Genes with progressively lower expression were involved in cardiac muscle tissue morphogenesis, muscle tissue morphogenesis, muscle organ morphogenesis, striated muscle contraction, and heart contraction. This suggests that monocytes/macrophages in MI patients have a negative effect on myocardial regeneration.

Through the NetworkAnalyst database, we obtained 9 TFs associated with hub genes. The heatmap in Figure 11B shows the trend of the expression of these TFs. PPARG, STAT3, RUNX2, and FOS had higher expression in MI patients. ceBPB, NR3C1, FOXC1, JUND, and FRDM1 had lower expression in MI patients.

## 4. Discussion

Under pathological conditions, different forms of cell death interact extensively and often in mixed forms.^[48]^ PANoptosis was found to be a mixed form of PCD that includes apoptosis, pyroptosis, and necroptosis.^[49]^ Studies have found that PANoptosis may be associated with a variety of diseases,^[50–52]^ but there are few studies related to MI. Exploring the role of PANoptosis in MI may provide new and feasible directions for the prevention and the treatment of MI.

In our study, 3 PRDEGs were identified including IL-1β, TLR4, and NLRP3. The 3 genes and corresponding components are closely linked in inflammation. IL-1β is an essential cytokine that mediates a variety of immune responses.^[53]^ TLR4 is a kind of membrane-bound pattern-recognition receptors which promotes IL-1β secretion and activation by recognizing pattern pathogen associated molecular patterns or damage associated molecular patterns.^[54]^ It has been shown that in vivo the microbes can activate the TLR4 signaling pathway subsequently recruiting NLRP3 inflammasome to induce IL-1β expression.^[55]^ NLRP3 has also been shown to mediate the cleavage of pro-IL-1β by caspase-1 to generate biologically active IL-1β in response to pathogen infection or cell injury.^[54,56]^ IL-1β inhibitors reduce the odds of recurrent infarction in patients with MI, according to a controlled clinical study.^[57]^ Activation of NLRP3 inflammasome has been proven to potentially promote progression of atherosclerosis,^[58]^ which is thought to be the underlying pathology of MI.^[59]^ The NF-κB signaling pathway was identified in the enrichment analysis of 3 genes. It has been widely reported that the TLR4/NF-κB signaling pathway is connected with inflammation and myocardial fibrosis in MI.^[60–62]^ Three genes are associated with the inflammatory response in MI, but how they are involved in PANoptosis in MI is unclear.

The caspase-1, activated by NLRP3 inflammasome, cleaves gasdermin D (GSDMD) to induce pyroptosis, resulting in the release of inflammatory factors.^[63]^ Some intrinsic apoptotic effectors, such as BAX and BAK, induce the activation of NLRP3 inflammasome and the secretion of IL-1β.^[64]^ Extrinsic apoptosis triggered by transmembrane receptors, including TLR4, has been reported to regulate the activation of NLRP3 inflammasome through multiple pathways.^[65,66]^ TLR4 activated by lipopolysaccharide (LPS) and pathogen associated molecular patterns can trigger necroptosis through activation of RIPK3.^[67]^ In conclusion, in the crosstalk of 3 PCDs, TLR4 often activates NLRP3 inflammasome and thus IL-1β. PANoptosis in disease is now found to be mostly virus-induced,^[11,68]^ but also synergistically induced by multiple cytokines.^[49]^ The systemic inflammatory response after MI may be a favorable condition for the induction of PANoptosis, which in turn further activates NLRP3 and IL-1β.

In our study, macrophages showed a higher correlation with the 3 genes. Macrophages in the healthy heart remove mitochondria secreted by cardiomyocytes to maintain cardiac homeostasis.^[69]^ In patients with MI, macrophages are involved in multiple stages of atherosclerosis from formation to rupture.^[70]^ In studies of PANoptosis, virally or bacterially infected macrophages are the classical model.^[71]^ Our study shows that macrophages may be the major cells involved in PANoptosis after MI.

Macrophages in MI patients and controls were in different states in the pseudotime analysis. It was shown that NLRP3 promotes M1 macrophage polarization and IL-1β production.^[72]^ Macrophages in MI patients were more likely to be of a proinflammatory phenotype. Compared with controls, macrophages from MI patients were less involved in the biological process of cardiac muscle tissue morphogenesis. Macrophage subpopulations in zebrafish are crucial in cardiac scar resolution, which may promote myocardial regeneration.^[73]^ Neonatal macrophages promote cardiomyocyte proliferation through the oncostatin M and gp130 pathways,^[74]^ but this fails to activate the regeneration of the adult heart after MI.^[75,76]^ More research is needed on how macrophages play a role in promoting cardiac regeneration in adults.

The drug-gene interaction network identified several repurposable candidates that could disrupt PANoptosis signaling. However, our ceRNA analysis cautions against simplistic miRNA-based interventions: competing lncRNA interactionsmay necessitate combinatorial targeting strategies. These insights align with clinical trial data showing that broad anti-inflammatory approaches outperform single-cytokine inhibition in reducing cardiovascular events, underscoring the need for PANoptosis-specific therapeutics.

There are several limitations in this study. Bulk RNA-seq cannot resolve dynamic PANoptosis activation during MI progression. Spatial transcriptomics could map cell death foci within infarct border zones. Functional validation in PANoptosis-deficient animal models is needed to confirm the mechanistic role of NLRP3, IL-1β, and TLR4. The AUC values of identified biomarkers (0.74–0.76) indicate moderate diagnostic utility, necessitating refinement through proteomic validation. Future studies should explore whether PANoptosis inhibition preserves cardiomyocytes post-MI, potentially synergizing with regenerative therapies targeting macrophage polarization.

## 5. Conclusion

This study shows that PANoptosis is associated with MI. Aberrant expression of NLRP3, IL-1β, and TLR4 in macrophages is an intrinsic link between PANoptosis and MI.

## Acknowledgments

Thank the researchers for sharing the data used in this study, and the participants included in the public dataset. Thank the GEO datasets for providing the data.

## Author contributions

**Conceptualization:** Pengfei Nie.

**Data curation:** Dongdong Que.

**Methodology:** Dongdong Que.

**Software:** Dongdong Que.

**Supervision:** Pengfei Nie.

**Writing – original draft:** Dongdong Que.

**Writing – review & editing:** Pengfei Nie.

## Supplementary Material


