# Supplementary material for: Identification of PANoptosis-related biomarkers in myocardial infarction via bioinformatics and single-cell analyses
Source: Medicine (Baltimore). 2026 Jan 23;105(4):e46750. doi: 10.1097/MD.0000000000046750 (PMC12851773; doi:10.1097/MD.0000000000046750)
Supplement: Supplementary file 1 [file medi-105-e46750-s001.pdf]

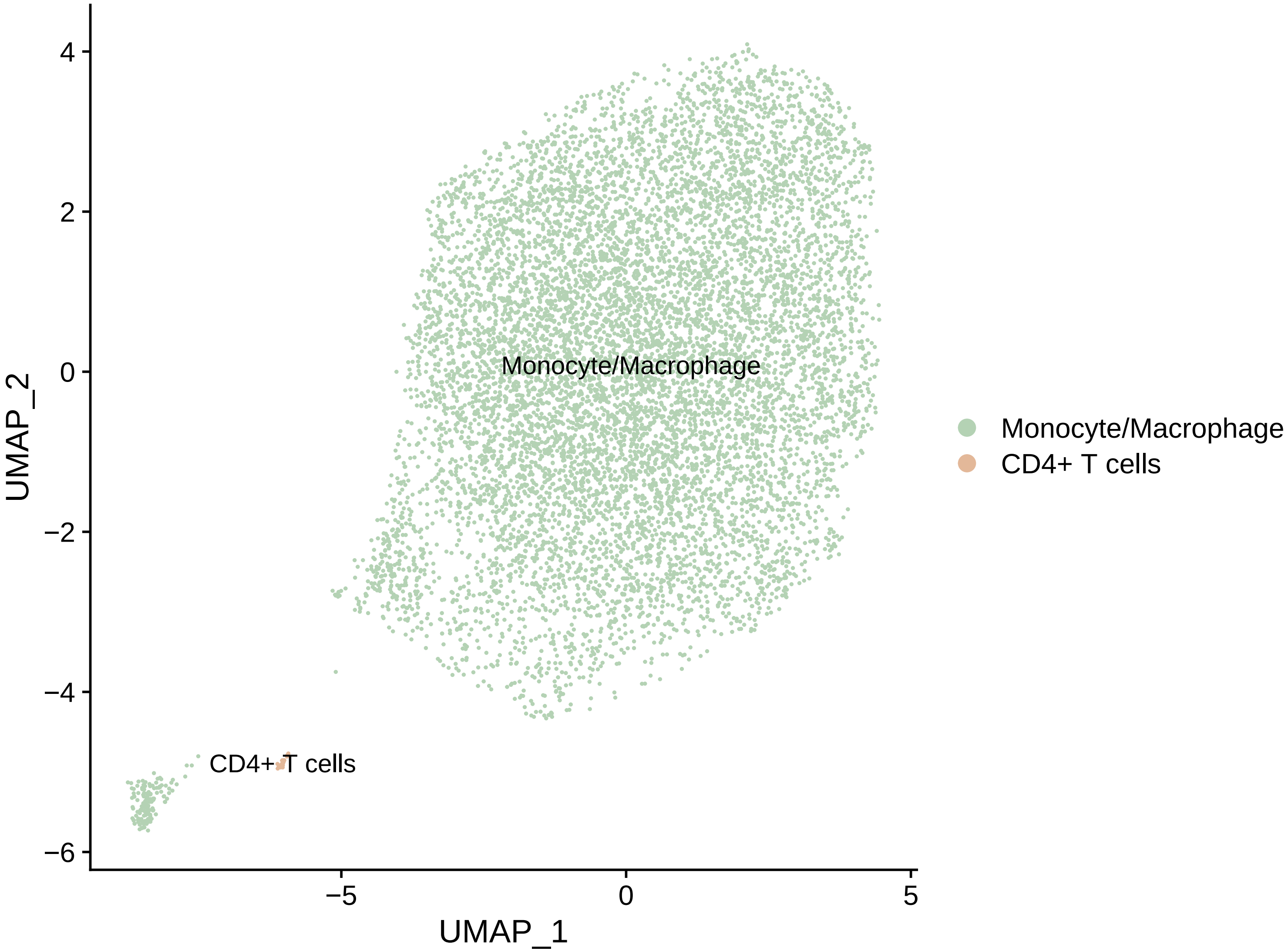

Monocyte/Macrophage
CD4+ T cells
UMAP_2
UMAP_1
4
2
0
−2
−4
−6
−5
0
5
Monocyte/Macrophage
CD4+ T cells

Supplementary Table 1

Datasets	Platform	Participants	Species	Tissues
GSE48060	GPL570	31 MI and 21 controls		Peripheral blood
GSE60993	GPL6884	17 MI and 7 controls		Peripheral blood
GSE61144	GPL6106	14 MI and 10 controls		Peripheral blood
GSE66360	GPL570	21 MI and 22 controls	*Homo sapiens*	Circulating endothelial cells
MI single-cell RNA-sequencing	CELLxGENE database	12 MI samples and 4 normal samples		Myocardium

161 DEGs of monocytes/macrophages from MI patients and controls
KAZN
EBF1
LENG8
SDS
ZNF331
FKBP5
ITGAX
GLUL
POSTN
SERPINE1
SPP1
TPST1
COL5A2
COL4A1
FAM20C
CALD1
PITPNC1
FN1
CCND3
KIF26B
GPC6
COL3A1
COL5A1
COL1A1
COL1A2
COL6A3
RORA
ZBTB16
BICC1
WWTR1
PDE4B
TRDN.AS1
PHACTR1
SLC8A1
VAV3
SLC16A7
FGF12
SORBS2
CDIN1
PXDNL
RYR2
MLIP
CTNNA3
TECRL
HRH1
ME1
LINC01374
WWP1
PDGFC
FRMD4B
MS4A6A
MS4A4E
KHDRBS2
ITPR2
DAB2
MAN1A1
PDE4D
SELENOP
MS4A4A
ARHGAP24
CCDC141
P2RY14
MAMDC2

SCN9A
RNF130
RNF150
ELMO1
ARHGAP18
CIITA
LYVE1
SIPA1L1
EPB41L3
TGFBI
PTPRC
B2M
LINC00278
EDA
CARMIL1
IL10RA
MVB12B
MCTP1
SPRED1
F13A1
IGSF21
ADAM28
FGF13
SUMF1
FLI1
SLC9A9
THRB
PID1
CPED1
WDFY2
GNG2
NR3C1
SYK
ACTC1
FPR1
ITM2B
FILIP1L
N4BP2L2
FPR3
SNX2
SNCA
COLEC12
STARD13
AFF3
CCDC91
ARHGAP6
MEF2C
TRPS1
CPVL
VOPP1
ST8SIA4
CD74
WIPF1
STX7
MSR1
HLA.DRB1
PLCL2
ANKRD44
MARCHF1
RB1
ARHGAP15
TANK
HIF1A.AS3
MIR99AHG

HDAC9
MB
RTN1
TCAP
SLC8A1.AS1
COX1
DTNA
MBNL1
COMMD10
FCGR2A
TLR2
ZFP36L1
MAP3K1
ATP8B4
GNAQ
QKI
RBPJ
MOB3B
MYH7
TNNI3
S100A6
CST3
COX3
ATP6
DCN
ANKUB1
CFD
MYL2
RPLP1
ENTPD1.AS1
MGP
GSN
TMSB4X
DLEU1
